# Practical Protocol for Making Calibration Curves for Direct and Sensitive Quantitative LC Orbitrap-MS of Large Neuropeptides

**DOI:** 10.5702/massspectrometry.A0087

**Published:** 2020-08-03

**Authors:** Tohru Yamagaki, Takashi Yamazaki

**Affiliations:** 1 Suntory Institute for Bioorganic Research, Suntory Foundation for Life Sciences

**Keywords:** neuropeptide Y, quantitative LC-MS, calibration curve, adsorption

## Abstract

Peptides larger than 3–4 kDa, such as neuropeptide Y (NPY), orexin-B, and alpha-MSH, have practical issues that arise when conducting direct and sensitive quantitative liquid chromatography (LC) orbitrap-FT mass spectrometry (MS) due to their adsorption and low ionization efficiency, especially in standard solutions. A mixing solvent consisting of 0.5% trifluoroacetic acid (TFA) and 35–50% aq. acetonitrile was developed as the standard NPY for creating calibration curves, as well as a matrix to block the experimental tube surface to minimize adsorption. The mixture matrix effectively blocked non-specific adsorption of the standard peptides with tryptic digested bovine serum albumin (BSA) (small fragment peptides) and orexin-B (a large chain peptide). A sample containing 1 : 100 peptide:water was detected in the developed sample solution. Finally, 2 to 1,000 fmol/μL NPY could be analyzed quantitatively and reproducibly using conventional LC-MS. Parameters of the calibration curves, such as X-intercept, Bias (%), and relative standard deviation (RSD), were adjusted to optimize the sample solutions and the sensitive and quantitative LC-MS analyses.

## INTRODUCTION

Neuropeptides are found in brain and central nervous system tissues and act as neurotransmitters and neurohormones. For example, neuropeptide Y (NPY), orexin-B, and α-MSH help regulate feeding behavior and control blood pressure.^1–3)^ The present report describes the development of improved practical protocols for producing the calibration curves for sensitive quantitative liquid chromatography-mass spectrometry (LC-MS) analysis of neuropeptides.

Calibration curves are important for sensitive quantitative LC-MS analyses. The experimental quality depends on the accuracy, precision, and detection limits of the calibration curve. In general, the amounts of biomolecules are so small that practical difficulties are encountered during quantitative analyses. Trace quantity analysis should be developed initially, followed by methods for experimental reproducibility and accuracy. If large amounts of bio-samples and target molecules are available, large-scale analyses can be performed under the exact and ideal calibration curves. Finally, quantitative LC-MS analyses of the biomolecules depend on the balance among sample availability, sensitivity, and reproducibility of the practical LC-MS system.

The goal of the present study was to quantify small amounts of neuropeptides in brain tissue. These neuropeptides exist in very small quantities in brain tissue. The strategy for developing a sensitive quantitative LC-MS protocol involved optimization of the calibration curves parameters of X-intercept, Bias (%), and relative standard deviation. To reduce the LC-MS detection limits, X-intercept and Bias (%) values can be used to evaluate the experimental conditions. Relative standard deviation represents the reproducibility of the experiments. The LC-MS experiment parameters and preparation protocols were optimized for the sensitive quantitative LC-MS analysis of neuropeptides.

Neuropeptide Y (NPY) is amidated at the C-terminal and contains 36 amino acids larger than 4 kDa.^4)^ Neuropeptides are expressed as precursor proteins that have no biological activity themselves. After the precursor proteins are cleaved and amidated enzymatically as post-translations, the bioactive peptides are generated.^5)^ To study the maturation process of the neuropeptides, precursor peptides and their intermediates in each maturation step need to be detected and analyzed directly without artificial enzymatic digestion.

The NPY is soluble in water because NPY contains basic amino acids and functions as a secretory hormone. However, NPY exhibits carry-over in the LC-MS. A previous report described how to troubleshoot the NPY carry-over in an LC-MS system.^6)^ In sensitive LC-MS analysis of NPY and neuropeptides larger than 3 or 4 kDa, creating calibration curves is difficult due to the neuropeptide chemical properties. Large neuropeptides were speculated to form multimers or clusters easily in an aqueous standard solution, inducing non-specific adsorption to the experimental tube surface and reducing their ionization efficiency. Thus, the sensitive quantitative LC-MS analysis of NPY as a neuropeptide was optimized by focusing on the contents of the preparation solutions and matrix solutions.

## EXPERIMENTAL

### Methods and materials

**Figure 1:**
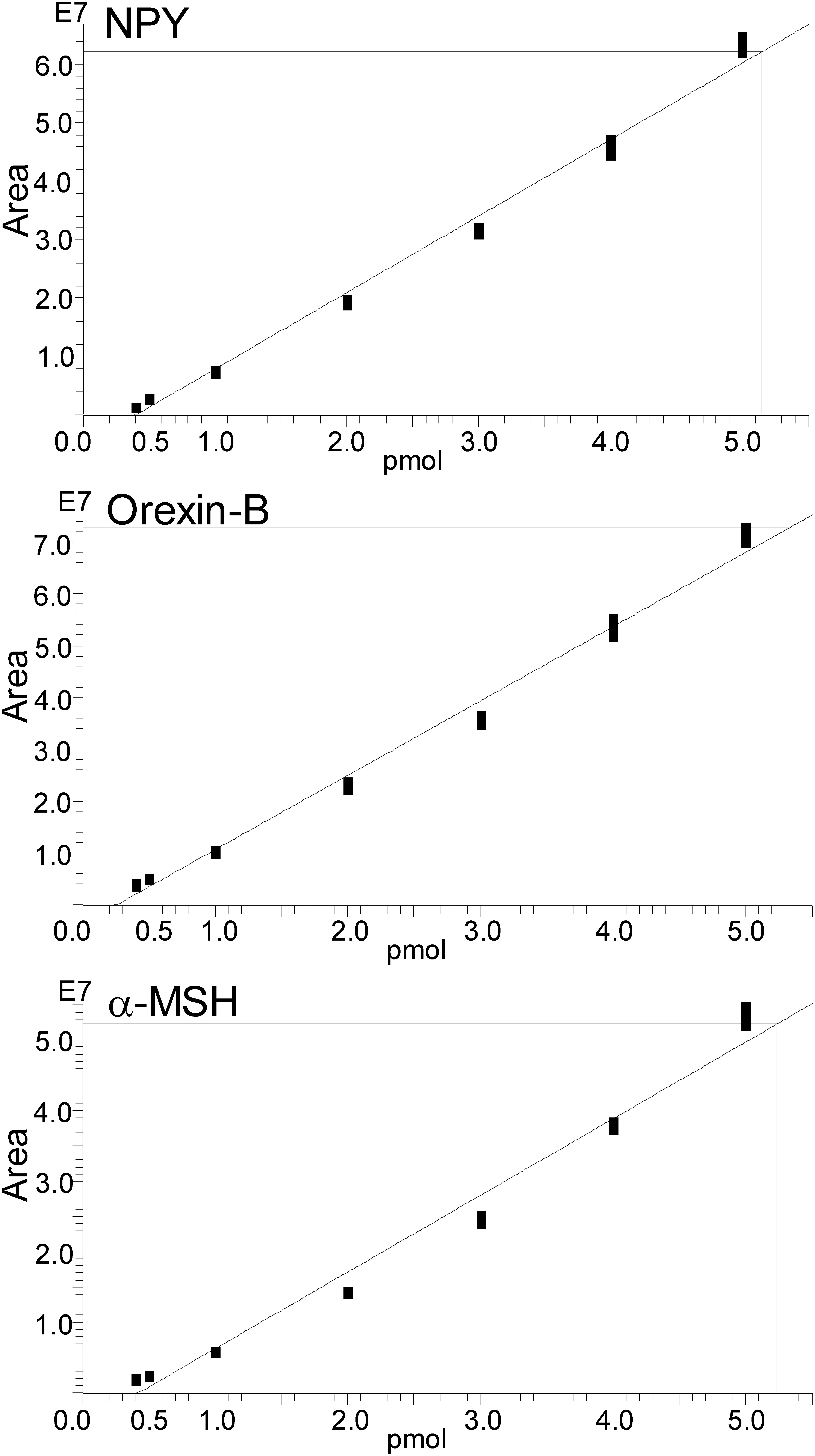
Fig. 1. Calibration curves of NPY, orexin-B, and α-MSH.

Neuropeptide Y (Peptide Institute, Inc., Osaka, Japan), trypsin-digested bovine serum albumin (BSA) MS standard (carboxymethyl-modified) (New England BioLabs, Inc., MA, USA), and HPLC grade acetonitrile (Nakalai Tesque, Inc., Kyoto, Japan) were used. MilliQ water (Merck Millipore, MA, USA) was used for all solutions. An aqueous solution containing 10.0 μM of NPY, orexin-B, and α-MSH was prepared as the stock solution. A series of 0.4, 0.5, 1.0, 2.0, 3.0, 4.0, and 5.0 μM standard samples were prepared from the stock solution in the calibration curves of Fig. 1, Table 1, and SI-1–3.

NPY X-intercept is 0.4012 pmol, orexin-B X-intercept is 0.2484 pmol, α-MSH X-intercept is 0.4251 pmol.

**Table 1:** Table 1. The LC experimental conditions.

Entry	Initial (B%)	B%	Gradient duration time (min)	Gradient ratio (B%/min)	Flow rate (μL/min)	Column type	FT-MS resolution
Figure 1	10	50	27	1.5	300	C18	120,000
Entry 1	10	40	10	3.0	200	C8	120,000
Entry 2	10	40	10	3.0	200	C8	120,000
Entry 3	10	45	10	3.5	200	C8	120,000
Entry 4	10	45	10	3.5	200	C8	120,000
Entry 5	10	45	10	3.5	200	C8	120,000
Entry 6	10	45	20	1.8	200	C8	120,000
Entry 7	10	40	25	1.2	200	C8	120,000
Entry 8	10	40	25	1.2	200	C8	120,000
Entry 9	10	35	21	1.2	200	C8	120,000
Entry 10	10	35	21	1.2	200	C8	120,000

The experiments of entry 1–10, the trypsin-digested BSA was diluted with water at 10 fmol/μL concentration as a basic matrix solution. When the various matrix solutions were made in entry 1–10, the pipette tips were washed gently with the digested BSA basic matrix solution over five times. When the series of the standard samples were made in entry 1–10, the vials and pipette tips were washed gently with the corresponding matrix solutions to block the surface of the vials and pipette tips before starting the experiments. The series of the standard samples were diluted with serial dilution due to the possibility of the sample adsorption to the vials and pipette tips.

### LC-MS

A Nexera UPLC/HPLC system (Shimadzu Co., Kyoto, Japan) was used in conjunction with a MS instrument. The sample-needle washing solution was 50% aq. acetonitrile in the auto-sampler. Aliquots of 1 μL of the standard and sample solutions were injected for the LC-MS experiments. An Aeris Peptide XB-C18 (ID 2.1 mm×250 mm, 2.6 μm) with a Security Guard Ultra guard column (Phenomenex Inc., CA, USA) was used at a flow rate of 0.3 mL/min. A YMC-Triart C8 metal-free column (ID 2.1 mm×250 mm, 2.6 μm) was used at a flow rate of 0.2 mL/min. The gradient elution solutions used were 0.1% (v/v) formic acid/water (solution A) and 0.1% (v/v) formic acid/acetonitrile (solution B). The gradient programs are summarized in Table 1.

All MS data were acquired on a Fourier transform (FT) orbitrap linear ion-trap hybrid (FT-Orbitrap Elite) MS instrument (Thermo Fisher Scientific, Inc., MA, USA). The experimental parameters were: ion-source of ESI, positive-ion mode detection, MS resolution of 120,000 with *m*/*z* 350–2,000 mass range, capillary temperature of 250°C, source heater temperature of 500°C, sheath gas flow rate of 50 (arbitrary unit specific to device), and AUX gas flow rate of 15 (arbitrary unit specific to device). The calibration curves were created using Xcalibur and QuanBrowser software of the FT-MS instrument (Thermo Fisher Scientific, Inc., MA, USA). The peak area of the most abundant peak was used for their calibration curves. The target peak of NPY was at *m*/*z*=712.8547 ([M+6H]^6+^); that of orexin-B was at *m*/*z*=734.9128 ([M+4H]^4+^); that of α-MSH was at *m*/*z*=555.6055 ([M+3H]^3+^).

## RESULTS AND DISCUSSIONS

Calibration curves are shown in Fig. 1. All the experimental raw data were summarized in supporting information 1–13 (SI-1–13).

### Evaluation of calibration curve parameters

#### Correlation coefficient (R) and coefficient of determination (R^2^)

Correlation coefficient (R) and coefficient of determination (R^2^) show the linearity of the calibration curves, and values of R and R^2^ greater than 0.998 are desirable for quantitative analysis, with values greater than 0.9998 for exact quantitative analysis. The R value for NPY was 0.99655 (Table 2), that for orexin-B was 0.99600 (SI-2), and that for α-MSH was 0.99165 (SI-3). Thus the linearity of the calibration curves was lower than the value required for the quantitative analysis in the concentration region of 0.4000–5.0000 pmol/μL.

**Table 2:** Table 2. The raw data of the calibration curve of NPY.

pmol	0.4000	0.5000	1.0000	2.0000	3.0000	4.0000	5.0000
run 1	1,341,435	2,901,846	7,563,526	19,659,526	32,465,846	46,129,607	64,582,131
run 2	1,289,424	2,784,674	7,854,728	20,018,785	32,636,601	46,428,878	65,958,699
run 3	1,292,378	2,834,302	7,606,358	20,048,353	32,041,437	48,535,814	64,734,702
run 4	1,309,210	2,784,646	7,629,543	20,482,323	32,162,329	48,622,286	66,918,689
run 5	1,314,321	2,740,069	7,445,986	20,312,752	32,988,855	48,155,686	64,329,921
Average	1309353.70	2809107.48	7620028.25	20104347.75	32459013.45	47574454.20	65304828.27
SD	20852.23	61638.00	149034.04	314094.02	379047.49	1199992.08	1098848.52
RSD (%)	1.59	2.19	1.96	1.56	1.17	2.52	1.68
SE	9325.40	27565.35	66650.05	140467.12	169515.19	536652.77	491420.00
Ideal value	5224386	6530483	13060966	26121931	39182897	52243863	65304828
Bias (%)	−74.94	−56.98	−41.66	−23.04	−17.16	−8.94	0.00
	Average	SD		Slope	13553325.23		
R	0.996556	0.937171		Y-intercept	−5473819.73		
R^2^	0.993124	0.878290		X-intercept	0.4039		

#### Standard deviation (SD) and relative standard deviation (RSD)

Standard deviation (SD) shows the variation in the experimental data, and relative standard deviation (RSD) is the SD divided by the average as a percentage. The RSD at 0.5000 pmol-NPY was 2.19% (Table 2), with good reproducibility.

#### X-intercept

Although an ideal calibration curve passes through the origin, the experimental X-intercepts were 0.3000–0.4000 pmol for the calibration curves of NPY, orexin-B, and α-MSH (Fig. 1, Table 2, SI-2, and SI-3). A quantitative detection limit less than 0.0100 pmol is required for analyzing 1.0-μL neuropeptide samples in mouse brain tissue. Therefore, more sensitive LC-MS protocols need to be developed.

#### Bias (%)

Bias (%) is the difference in the experimental average from the ideal values, calculated as follows: 
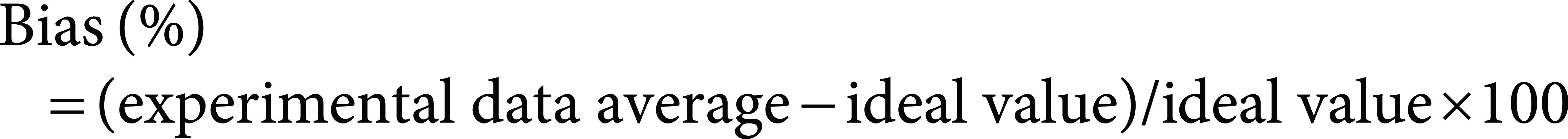
(1)

In general, Bias (%) values are about ±20% for quantitative analyses. The MS signals of the 5.0000-pmol samples were normalized as 100% of Bias, which caused the ideal calibration curve to pass through the origin. The Bias (%) values for the 2.0000-pmol samples of NPY, orexin-B, and α-MSH were approximately −20%; however, those of less concentrated samples were greatly reduced, especially NPY (Table 2, SI-1, and SI-2).

### Optimization strategy for sensitive qualitative LC-MS

Initially, the X-intercept and Bias (%) values were used to optimize the sensitivity of the LC-MS analysis of NPY by reducing the adsorption of a target peptide. A practical issue involving the sample solutions of large chain peptides, such as NPY, is that their Bias (%) value is greatly reduced at lower concentrations of the calibration curves (Fig. 1), which is thought to be due to the adsorption and aggregating properties of the peptides.

When an aliquot of the sample absorbs non-specifically to the inside surface of the experimental tube, the adsorption ratio for highly concentrated samples is relatively low or negligible. That ratio in low concentration samples is relatively high, resulting in loss of the analytical sample. Therefore, the detection signals of LC-MS were much smaller in the lower concentration samples, causing an increase in the X-intercept and a significant reduction in Bias (%). Thus, sample solution preparation is very important for suppression of non-specific adsorption in sensitive quantitative LC-MS analysis.

BSA is used as a blocking regent of non-specific adsorption of the analytes in biochemical experiments of proteins, such as immunohistochemistry and enzyme-linked immune sorbent assays (ELISA).^7,8)^ It has not been applied in reversed-phase liquid chromatography. Neuropeptide samples are applied to ODS or C-8 columns of LC systems, and so whole BSA cannot be used directly. Therefore, a tryptic-digested BSA fragment peptide mixture was used as the non-specific adsorption blocking matrix. All sample solutions in Table 3, except for entry 5, contained 10 fmol/μL tryptic-digested BSA fragment peptide mixture.

**Table 3:** Table 3. NPY calibration curve parameters in the various sample solutions.

Entry	Sample solution contents		Matrix peptide	Concentration range (pmol/μL)	Dynamic range	Column temp.(°C)	R^2^	X-intercept (pmol/μL)	Bias (%)^e)^	RSD^e)^	Data^g)^
	Aqueous content	Acetonitrile content									
1	Water	0	Digested BSA^c)^	0.1250–2.0000	16	40°C	0.98957	0.1821	−98.19	26.84	SI-4
2	0.1% FA^a)^	10%	Digested BSA	0.0625–2.0000	32	40°C	0.99830	−0.0325	−2.12	8.26	SI-5
3	0.5% TFA^b)^	10%	Digested BSA	0.0500–2.0000	40	40°C	0.99958	0.0455	−42.48	19.31	SI-6
4	0.5% TFA	50%	Digested BSA	0.0500–2.0000	40	40°C	0.99975	−0.0156	17.25	4.25	SI-7
5	0.5% TFA	50%	Digested BSA+Orexin-B (0.5)^d)^	0.0500–2.0000	40	40°C	0.99859	−0.0345	22.99	1.26	SI-8
6	0.1% TFA	35%	Digested BSA+Orexin-B (1.0)	0.0500–2.0000	40	70°C	0.99828	−0.0009	−7.24	2.77	SI-9^h)^
7	0.1% TFA	35%	Orexin-B (1.0)	0.0010–0.0200	20	70°C	0.81259	0.0026	−1.15	48.99^f)^	SI-10^i)^
8	0.1% TFA	35%	Digested BSA+Orexin-B (1.0)	0.0050–0.1000	20	70°C	0.99995	−0.0009	11.35	2.8	SI-11^h)^
9	0.1% TFA	35%	Digested BSA+Orexin-B (1.0)	0.0020–1.0000	500	70°C	0.99983	−0.0057	15.85	3.24	SI-12^h)^
10	0.1% TFA	35%	Digested BSA+Orexin-B (1.0)	0.0020–1.0000	500	70°C	0.99994	−0.0018	18.76	3.13	SI-13^h)^

^a)^ FA means formic acid, ^b)^ TFA means trifluoro acetic acid, ^c)^ digested BSA means 10 fmol/μL tryptic digested BSA fragment peptides, ^d)^ concentration of orexin-B (pmol/μL), ^e)^ the data at the lowest concentration, ^f)^ the data at 0.005 pmol/μL, ^g)^ all data are referenced in Supporting information, ^h)^ SI-6, 8–10 are the same experimental parameters, ^i)^ the matrix content of SI-7 is only orexin-B.

#### Overcoming carry-over in LC-MS systems

The carry-over of peptides in the LC-MS system hindered sensitive quantitative LC-MS analysis, because the last sample carried over and was detected in the next experiments. The carry-over of NPY in LC-MS analysis has been resolved previously using washing programs in the auto-sampler and LC lines, changing LC columns, and not using a guard column.^6)^ In this study, these measures were applied while developing the LC system.

#### Reversed-phase LC column

Aeris Peptide XB-C-18 was used for the peptide analyses (Fig. 1, Table 2, SI-2, and SI-3). We changed the column to that based on C-8 because of the peak shapes of eluted NPY showed tailing. The inner-column material was based on plastic to reduce carry-over in the YMC-Triart C8 metal-free column. All data in Table 3 were acquired using the metal-free C-8 column.

### Improving sample solution contents using acetonitrile, acids, and non-specific adsorption blocking matrix

Sample solutions and experimental conditions of NPY were improved (Table 3) to achieve sensitive quantitative LC-MS of neuropeptides. For entry 1, a series of sample solutions were prepared with water and matrix to create a calibration curve. The X-intercept of 0.1000 pmol was smaller than that of NPY (0.4000 pmol) (Table 2 and Fig. 1), suggesting that a digested BSA fragment peptide mixture acted effectively as a non-specific adsorption blocking matrix.

Solutions for entries 2–4 were prepared with acids and aqueous acetonitrile. In general, organic solvents are not used for samples in reversed-phase LC (RP-LC) because the samples might not be attracted to the RP-LC column. The NPY was thought to have high hydrophobicity because of its high adsorption. Therefore, an attempt was made to dissolve NPY in 100% acetonitrile, but the attempt was not successful because the NPY aggregated. Since NPY may have amphiphilicity, 10–50% acetonitrile was added. The injection volume was fixed at 1 μL, which is so small that the sample can be held on a C-8 RP-LC column. Formic acid or TFA also was added to the solution because of the many basic amino acid residues of NPY. Adding acetonitrile and acid effectively improved the X-intercept value and Bias (%) in entry 2 (0.1% formic acid/10% aqueous acetonitrile solution). After the improvements, addition of 0.5% TFA and a few tens of percent acetonitrile was selected for preparing the contents of organic solvent and acids in entries 3 and 4.

#### Reproducibility

In experimental runs 1–5 at the lowest concentration (Table 4, entry 1), the signal intensity of run 2 is much less than that of run 1. The NPY solution shown in entry 1 was only water (Table 2). In the aqueous solution of NPY, NPY dissolved at the first (run 1). During the wait period of run 2, the multimerization of NPY molecules proceeded gradually and NPY absorbed on the experimental tubes. The amount of adsorption of NPY depended on the experimental duration.

**Table 4:** Table 4. The raw data of the calibration curve of NPY in entry 1.

pmol	0.1250	0.2500	0.5000	1.0000	2.0000
run 1	64656	419698	5335556	20271071	37334270
run 2	44124	298397	5287572	21222916	38033965
run 3	34786	305973	5273383	19953304	40767359
run 4	38821	295382	4990088	19968446	41399218
run 5	38883	284644	5093212	19634758	38588179
Average	44253.79	320818.80	5195962.24	20210098.92	39224598.20
SD	11877.48	55802.81	147321.39	609276.82	1768124.80
RSD (%)	26.84	17.39	2.84	3.01	4.51
SE	5311.77	24955.78	65884.13	272476.88	790729.45
Ideal value	2451537	4903075	9806150	19612299	39224598
Bias (%)	−98.19	0.82	13.25	51.52	100.00
	Average		Slope	21923148.65	
R	0.994771		Y-intercept	−3991293.81	
R^2^	0.989569		X-intercept	0.1821	

Adding acetonitrile and acid in the solutions shown as entries 2–4 improved the experimental reproducibility as shown in Table 5 (entry 4 of Table 3) (see SI-5, SI-6, and SI-7). The addition of acetonitrile and acid resulted in effective of the adsorption and multimerization of NPY peptides. It is important for high reproducibility in sensitive LC-MS analysis that the samples dissolve well and are stable in solution for long experimental duration.

**Table 5:** Table 5. The raw data of the calibration curve of NPY in entry 4.

pmol	0.0500	0.2500	0.5000	1.0000	2.0000
run 1	5,958,059	26,290,241	49,976,892	106,203,465	191,836,528
run 2	5,646,309	25,771,693	47,908,604	98,950,655	191,066,184
run 3	5,757,907	26,579,344	49,424,093	97,028,111	196,022,792
run 4	5,729,540	25,133,530	49,593,576	100,864,203	193,003,463
run 5	5,229,032	24,545,452	48,239,358	96,182,477	194,254,474
Average	5664169.18	25664052.14	49028504.69	99845782.26	193236688.18
SD	240455.62	744872.77	806494.06	3567005.52	1762055.24
RSD (%)	4.25	2.90	1.64	3.57	0.91
SE	107535.02	333117.23	360675.11	1595213.36	788015.06
Ideal value	4830917	24154586	48309172	96618344	193236688
Bias (%)	17.25	6.25	1.49	3.34	0.00
	Average	SD		Slope	96299482.14
R	0.999872	0.555323		Y-intercept	1500232.86
R^2^	0.999745	0.308383		X-intercept	−0.0156

For the sample labeled as entry 4 (Table 3) at 0.0500 pmol/μL, the signals of runs 1–5 decreased gradually; however, NPY adsorption still proceeded slowly as shown in Table 5 and SI-7. Therefore, orexin-B was added to the matrix of digested BSA peptides (entry 5 of Table 3, Table 6, and SI-8). The signal reduction in runs 1–5 was suppressed by entry 5. Addition of orexin-B to the matrix solution effectively suppressed non-specific adsorption of NPY to the experimental tubes. The digested BSA fragment peptides were so small that the adsorption of NPY was hindered partly because the adsorption properties of NPY and digested BSA peptides were so different. The large peptide orexin-B 3 kDa competitively blocked the non-specific adsorption of NPY 4.3 kDa on the experimental tubes.

**Table 6:** Table 6. The raw data of the calibration curve of NPY in entry 5.

pmol	0.0500	0.2500	0.5000	1.0000	2.0000
run 1	5,627,869	25,172,972	49,120,680	96,396,203	182,219,944
run 2	5,746,266	25,756,127	50,189,079	98,538,027	182,803,507
run 3	5,556,831	25,774,547	49,067,571	98,474,773	182,251,890
run 4	5,577,659	24,620,331	49,188,634	100,848,603	181,806,813
run 5	5,695,441	25,371,713	48,848,730	99,381,324	188,180,298
Average	5640813.04	25339138.22	49282938.92	98727786.22	183452490.35
SD	71152.56	426337.27	467168.30	1446463.26	2385045.35
RSD (%)	1.26	1.68	0.95	1.47	1.30
SE	31820.39	190663.82	208924.02	646878.04	1066624.71
Ideal value	4586312	22931561	45863123	91726245	183452490
Bias (%)	22.99	10.50	7.46	7.63	0.00
	Average	SD		Slope	91237847.08
R	0.999296	0.988281		Y-intercept	3147869.57
R^2^	0.998592	0.976698		X-intercept	−0.0345

The matrix of entry 6 (Table 3) contained 1.0000 pmol/μL orexin-B and 10 fmol/μL tryptic digested BSA for improving the matrix solution. In addition, the column temperature was changed from 40 to 70°C. The acetonitrile concentration was adjusted from 50 to 35% because the peptides adhered to the column (Table 3). A comparison of entries 5 and 6 from Table 3 in SI-8 and SI-9, respectively showed a large improvement in the absolute value of the X-intercept (0.0009 is the lowest) and some improvement in Bias (%). The R^2^ and RSD values also were good (entries 5 and 6, Table 3).

In entry 7 (Table 3), the matrix contained only orexin-B without BSA fragments to evaluate the effect of digested BSA. The reproducibility of runs 1–5 for entry 7 (SI-10) at the lowest concentration decreased below that of entry 6 (SI-9) (Table 3). The RSD of entry 7 increased. Therefore, orexin-B alone was not adequate to block NPY adsorption and stabilize the NPY solution. Both orexin-B and digested BSA mixtures effectively blocked NPY adsorption and multimerization in the sample solutions, suggesting that small fragments of digested BSA hindered multimerization of NPY and stabilized the monomers, and that large orexin-B peptides blocked non-specific adsorption of NPY competitively on the tube surface.

Thus, entry 6 (Table 3) represented the best matrix solution content and the experimental conditions for the region from 0.0500 to 2.0000 pmol/μL (Table 3).

### Trace quantity concentration region analysis

Trace quantity samples entries 8 and 9 (Table 3) were analyzed using the same experimental conditions used for entry 6 (see SI-9, SI-11, and SI-12). The X-intercept and RSD values were small enough to allow quantitative analysis from 0.0020 to 1.0000 pmol/μL in the enlarged concentration range (Table 3). In addition, all of the HPLC stainless steel pipes were replaced with bio-inert pipes based on PEEK tubes for entry 10 analysis. The X-intercept and RSD values for entry 10 at the lowest concentration were lower than those for entry 9, as shown in Table 3. Thus, the sample solutions and sensitive quantitative LC-MS analysis protocols were optimized using calculation curve parameters from 0.0020 to 1.0000 pmol/μL.

## CONCLUSION

Enzymatic digested fragments of peptides and proteins usually are analyzed by LC-MS. These peptide fragment have greater ionization efficiency, no or much less adsorption, and no or much less self-aggregation compared to peptides larger than 3 or 4 kDa. Peptides larger than 4 kDa have lower ionization efficiency, and severe adsorption and self-aggregation issues in solution, which hinders reproducible and sensitive analyses. The experimental protocols developed here are essential for the analysis of large peptides. The sample preparation solution was an important factor in the sensitive quantitative LC-MS analysis of NPY. The sample solutions contained 0.5% TFA and some acetonitrile, and yet were analyzed by reversed-phase LC. The sample solutions also contained a non-specific adsorption blocking matrix with tryptic digested BSA fragment peptides and whole orexin-B. Two types of small and large peptides mixtures were very effective for stabilization of the solution sample and blockage of non-specific adsorption and self-aggregation. These results can be applied to improve analyses of other large peptides.

## Notes

Mass Spectrom (Tokyo) 2020; 9(1): A0087
