# Supplementary material for: Practical Protocol for Making Calibration Curves for Direct and Sensitive Quantitative LC Orbitrap-MS of Large Neuropeptides
Source: Mass Spectrom (Tokyo). 2020 Aug 3;9(1):A0087. doi: 10.5702/massspectrometry.A0087 (PMC7392841; doi:10.5702/massspectrometry.A0087)

Supporting Information

Practical Protocol for Making Calibration Curves for Direct and Sensitive Quantitative LC Orbitrap-MS of Large Neuropeptides.

Tohru Yamagaki and Takashi Yamazaki

SI-1. The raw data of the calibration curve of NPY in LC-MS using Aeris PEPTIDE XB-C18.
SI-2. The raw data of the calibration curve of orexin-B in LC-MS using Aeris PEPTIDE XB-C18.
SI-3. The raw data of the calibration curve of α-MSH in LC-MS using Aeris PEPTIDE XB-C18.
SI-4. The raw data of the calibration curve of entry 1 NPY in LC-MS using YMC-Triart C8.
SI-5. The raw data of the calibration curve of entry 2 NPY in LC-MS using YMC-Triart C8.
SI-6. The raw data of the calibration curve of entry 3 NPY in LC-MS using YMC-Triart C8.
SI-7. The raw data of the calibration curve of entry 4 NPY in LC-MS using YMC-Triart C8.
SI-8. The raw data of the calibration curve of entry 5 NPY in LC-MS using YMC-Triart C8.
SI-9. The raw data of the calibration curve of entry 6 NPY in LC-MS using YMC-Triart C8.
SI-10. The raw data of the calibration curve of entry 7 NPY in LC-MS using YMC-Triart C8.
SI-11. The raw data of the calibration curve of entry 8 NPY in LC-MS using YMC-Triart C8.
SI-12. The raw data of the calibration curve of entry 9 NPY in LC-MS using YMC-Triart C8.
SI-13. The raw data of the calibration curve of entry 10 NPY in LC-MS using YMC-Triart C8.

Supporting Information 1 (SI-1)

The raw data of the calibration curve of NPY in LC-MS using Aeris PEPTIDE XB-C18.
Sample solution: Water, mixture of NPY, a-MSH, Orixin-B (Aeris PEPTIDE, guard column)

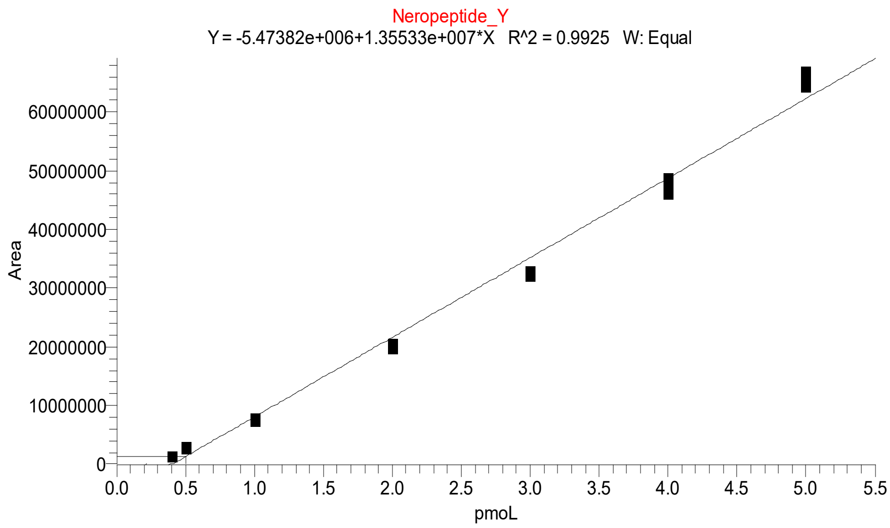

pmol	0.4000	0.5000	1.0000	2.0000	3.0000	4.0000	5.0000
run 1	1,341,435	2,901,846	7,563,526	19,659,526	32,465,846	46,129,607	64,582,131
run 2	1,289,424	2,784,674	7,854,728	20,018,785	32,636,601	46,428,878	65,958,699
run 3	1,292,378	2,834,302	7,606,358	20,048,353	32,041,437	48,535,814	64,734,702
run 4	1,309,210	2,784,646	7,629,543	20,482,323	32,162,329	48,622,286	66,918,689
run 5	1,314,321	2,740,069	7,445,986	20,312,752	32,988,855	48,155,686	64,329,921
Average	1309353.70	2809107.48	7620028.25	20104347.75	32459013.45	47574454.20	65304828.27
SD	20852.23	61638.00	149034.04	314094.02	379047.49	1199992.08	1098848.52
RSD(%)	1.59	2.19	1.96	1.56	1.17	2.52	1.68
SE	9325.40	27565.35	66650.05	140467.12	169515.19	536652.77	491420.00
Ideal value	5224386	6530483	13060966	26121931	39182897	52243863	65304828
Bias(%)	−74.94	−56.98	−41.66	−23.04	−17.16	−8.94	0.00

Slope	13553325.23
Y−intercept	−5473819.73
X−intercept	0.4039

	Average	SD
R	0.996556	0.937171
R^2	0.993124	0.878290

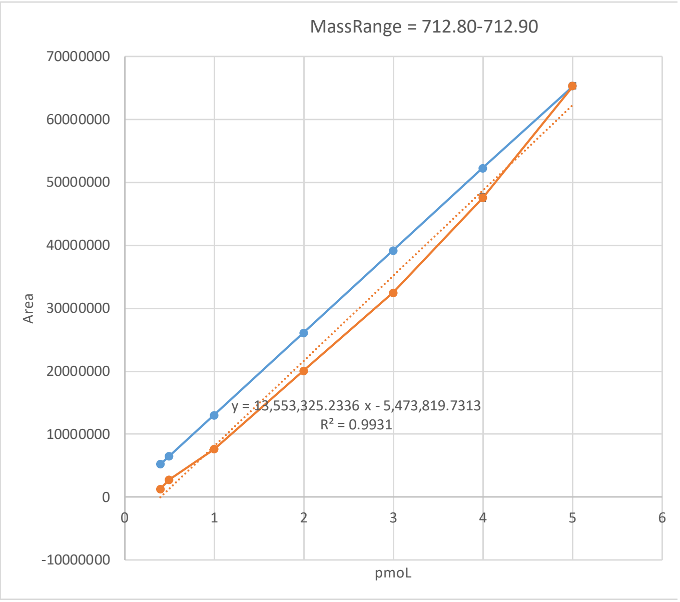

Supporting Information 2 (SI-2)

The raw data of the calibration curve of orexin-B in LC-MS using Aeris PEPTIDE XB-C18.　Sample solution: Water, mixture of NPY, a-MSH, Orixin-B (Aeris PEPTIDE, guard column)

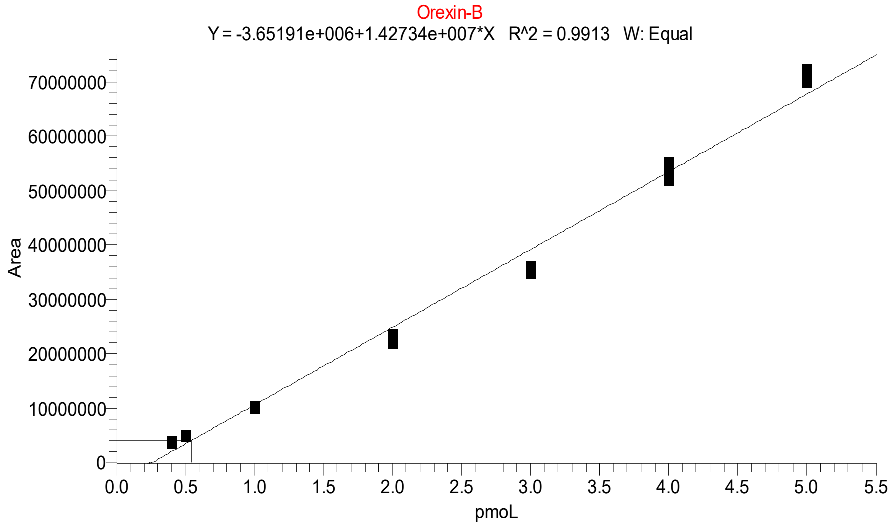

pmol	0.4000	0.5000	1.0000	2.0000	3.0000	4.0000	5.0000
run 1	4,055,032	5,096,303	10,093,774	23,592,299	36,212,197	52,769,599	70,522,551
run 2	3,955,575	4,923,906	10,471,039	22,367,738	34,852,791	52,023,966	70,815,906
run 3	3,804,094	5,032,609	10,240,454	23,094,648	35,843,115	51,918,414	69,963,579
run 4	3,750,105	4,903,866	10,375,972	22,677,149	35,464,445	53,842,121	69,837,622
run 5	3,689,599	4,928,897	10,447,858	22,066,615	35,771,659	55,158,219	72,351,032
Average	3850880.75	4977116.29	10325819.56	22759689.73	35628841.32	53142464.10	70698137.81
SD	150826.86	83377.21	157820.89	601295.83	508894.35	1363923.87	1007077.71
RSD(%)	3.92	1.68	1.53	2.64	1.43	2.57	1.42
SE	67451.82	37287.42	70579.65	268907.67	227584.47	609965.30	450378.85
Ideal value	5655851	7069814	14139628	28279255	42418883	56558510	70698138
Bias(%)	-31.91	-29.60	-26.97	-19.52	-16.01	-6.04	0.00

Slope	14273355.21
Y-intercept	-3651914.03
X-intercept	0.2559

	Average	SD
R	0.996004	0.898851
R^2	0.992024	0.807933

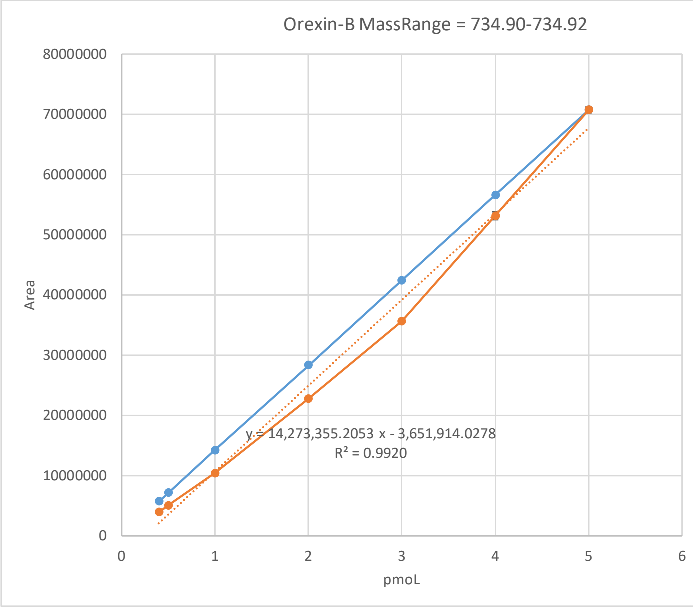

Supporting Information 3 (SI-3)

The raw data of the calibration curve of α-MSH in LC-MS using Aeris PEPTIDE XB-C18. Sample solution: Water, mixture of NPY, α-MSH, Orixin-B (Aeris PEPTIDE, guard column)

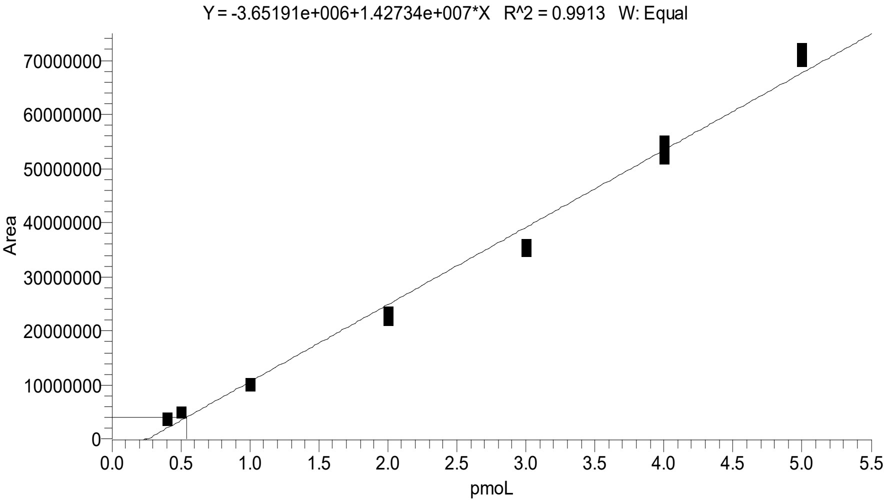

pmol	0.4000	0.5000	1.0000	2.0000	3.0000	4.0000	5.0000
run 1	2,037,776	2,467,834	5,674,732	14,045,454	23,942,187	38,277,026	52,168,674
run 2	1,952,445	2,443,986	5,913,203	14,279,518	24,954,595	37,370,806	53,730,346
run 3	1,969,029	2,398,768	5,757,308	14,296,796	25,079,932	37,908,952	54,782,463
run 4	1,885,401	2,503,451	5,737,190	14,196,083	25,092,564	38,399,532	52,598,627
run 5	1,941,853	2,493,840	5,842,637	14,309,877	24,900,899	38,481,932	52,256,280
Average	1957300.83	2461575.88	5785014.08	14225545.81	24794035.31	38087649.55	53107278.10
SD	54876.22	42094.78	93500.74	110002.80	483141.26	456724.68	1124539.82
RSD(%)	2.80	1.71	1.62	0.77	1.95	1.20	2.12
SE	24541.39	18825.36	41814.80	49194.75	216067.34	204253.49	502909.50
Ideal value	4248582	5310728	10621456	21242911	31864367	42485822	53107278
Bias(%)	−53.93	−53.65	−45.53	−33.03	−22.19	−10.35	0.00

Slope	10861689.67
Y-intercept	−4611780.89
X-intercept	0.4246

	Average	SD
R	0.991651	0.915256
R^2	0.983372	0.837693

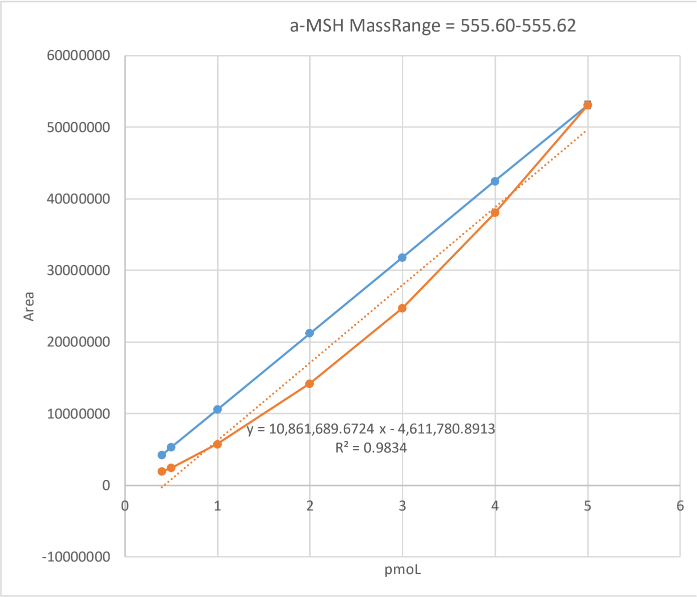

Supporting Information 4 (SI-4)

The raw data of the calibration curve of entry 1 NPY in LC-MS using YMC-Triart C8.

Sample solution: Water, Matrix: 10 fmol/µL digested BSA

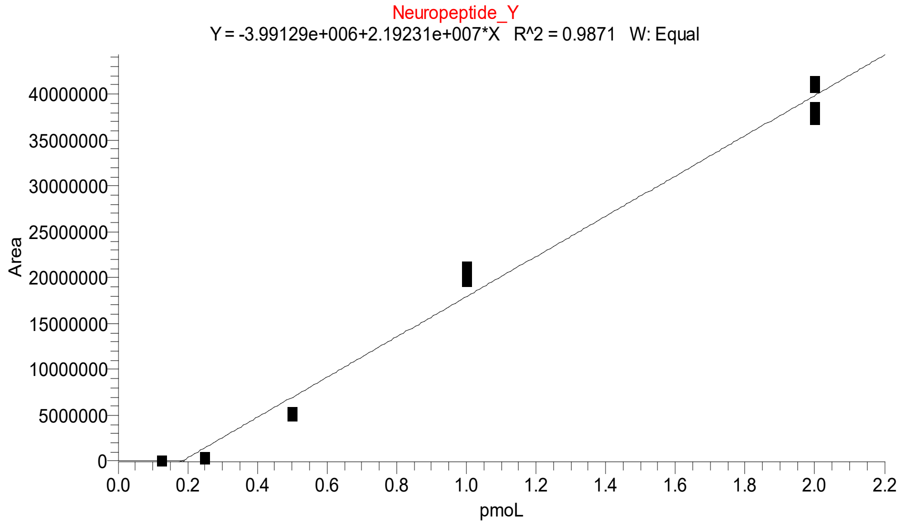

pmol	0.1250	0.2500	0.5000	1.0000	2.0000
run 1	64656	419698	5335556	20271071	37334270
run 2	44124	298397	5287572	21222916	38033965
run 3	34786	305973	5273383	19953304	40767359
run 4	38821	295382	4990088	19968446	41399218
run 5	38883	284644	5093212	19634758	38588179
Average	44253.79	320818.80	5195962.24	20210098.92	39224598.20
SD	11877.48	55802.81	147321.39	609276.82	1768124.80
RSD(%)	26.84	17.39	2.84	3.01	4.51
SE	5311.77	24955.78	65884.13	272476.88	790729.45
Ideal value	2451537	4903075	9806150	19612299	39224598
Bias(%)	−98.19	0.82	13.25	51.52	100.00

Slope	21923148.65
Y−intercept	−3991293.81
X−intercept	0.1821

	Average
R	0.994771
R^2	0.989569

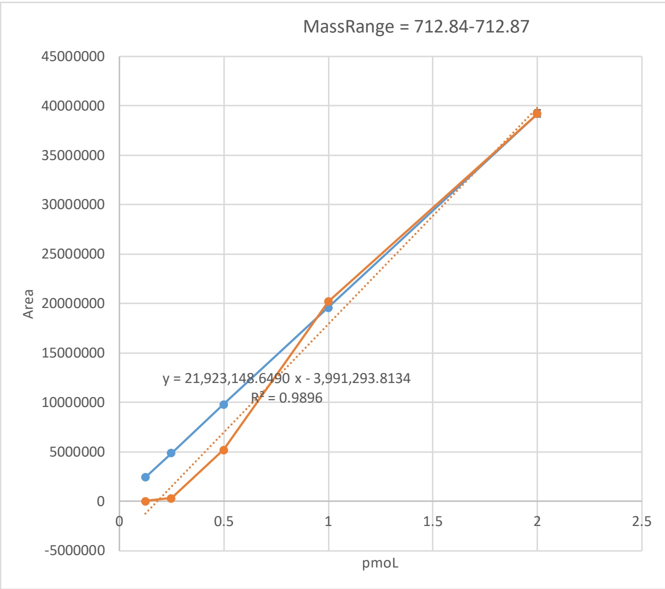

Supporting Information 5 (SI-5)

The raw data of the calibration curve of entry 2 NPY in LC-MS using YMC-Triart C8.

Sample solution: 10% acetonitrile/0.1% formic acid, Matrix: 10 fmol/µL digested BSA

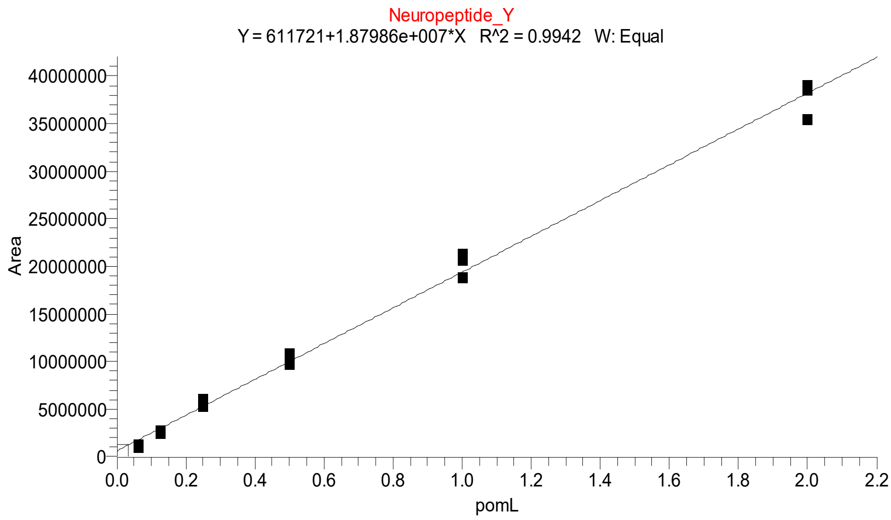

pmol	0.0625	0.1250	0.2500	0.5000	1.0000	2.0000
run 1	1,262,244	2,789,719	6,143,919	10,083,071	21,297,180	38,543,232
run 2	1,115,733	2,791,829	5,346,838	10,832,501	18,840,279	39,101,495
run 3	1,082,621	2,477,629	5,377,362	9,738,407	20,755,375	35,490,192
Average	1153532.87	2686392.36	5622706.26	10217993.21	20297611.25	37711639.62
SD	95590.96	180797.33	451641.19	559386.76	1290833.60	1943974.66
RSD(%)	8.29	6.73	8.03	5.47	6.36	5.15
SE	55189.47	104383.39	260755.16	322962.09	745263.13	1122354.29
Ideal value	1178489	2356977	4713955	9427910	18855820	37711640
Bias(%)	−2.12	13.98	19.28	8.38	7.65	0.00

Slope	18798615.90
Y-intercept	611720.91
X-intercept	−0.0325

	Average	SD
R	0.999151	0.985592
R^2	0.998303	0.971392

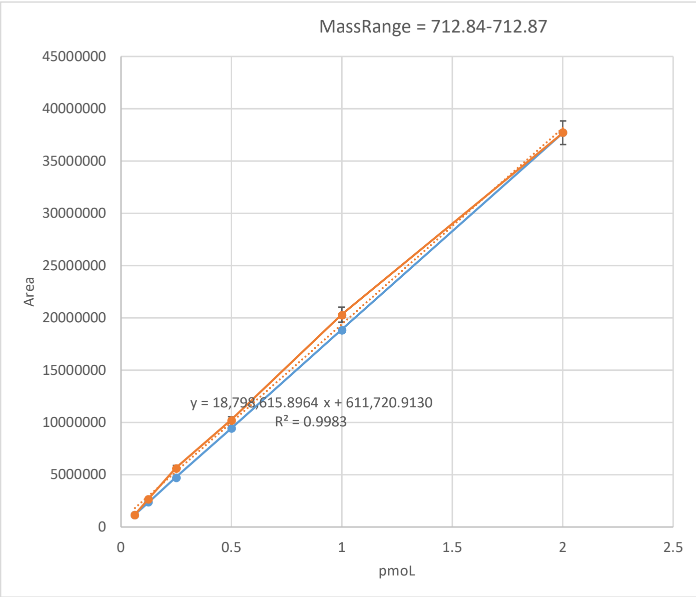

Supporting Information 6 (SI-6)

The raw data of the calibration curve of entry 3 NPY in LC-MS using YMC-Triart C8.
Sample solution: 10% acetonitrile/0.5% TFA, Matrix: 10 fmol/μL digested BSA

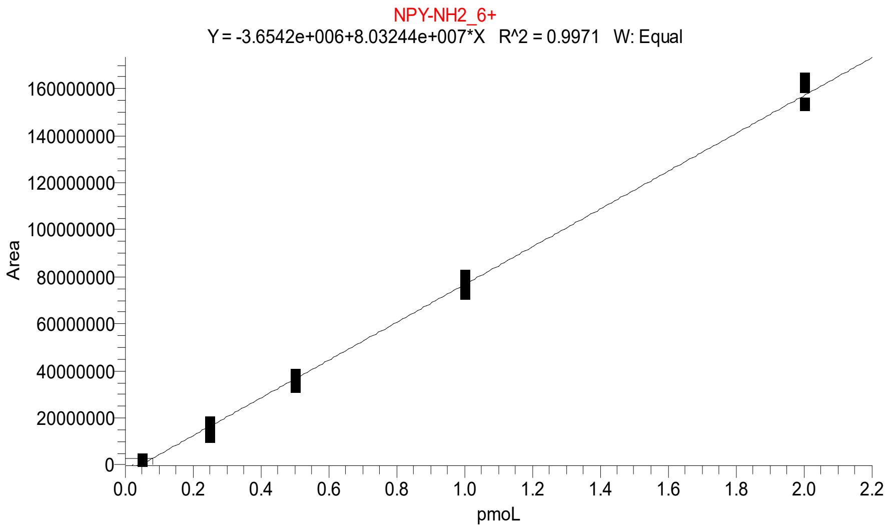

pmol	0.0500	0.2500	0.5000	1.0000	2.0000
run 1	2,853,408	18,419,533	38,880,779	80,752,778	164,815,438
run 2	2,520,070	16,526,902	37,074,404	78,538,400	160,656,714
run 3	2,227,012	14,626,389	34,704,582	75,650,437	154,069,939
run 4	1,930,919	13,439,358	34,210,881	76,425,706	152,780,798
run 5	1,776,234	11,951,007	33,208,992	72,704,156	154,064,432
Average	2261528.84	14992637.75	35615927.69	76814295.34	157277464.21
SD	436798.09	2544401.05	2312166.28	3036568.78	5221880.94
RSD(%)	19.31	16.97	6.49	3.95	3.32
SE	195342.04	1137890.74	1034032.19	1357994.84	2335296.15
Ideal value	3931937	19659683	39319366	78638732	157277464
Bias(%)	−42.48	−23.74	−9.42	−2.32	0.00

Slope	80324439.46
Y-intercept	−3654203.22
X-intercept	0.0455

	Average	SD
R	0.999791	0.934593
R^2	0.999581	0.873464

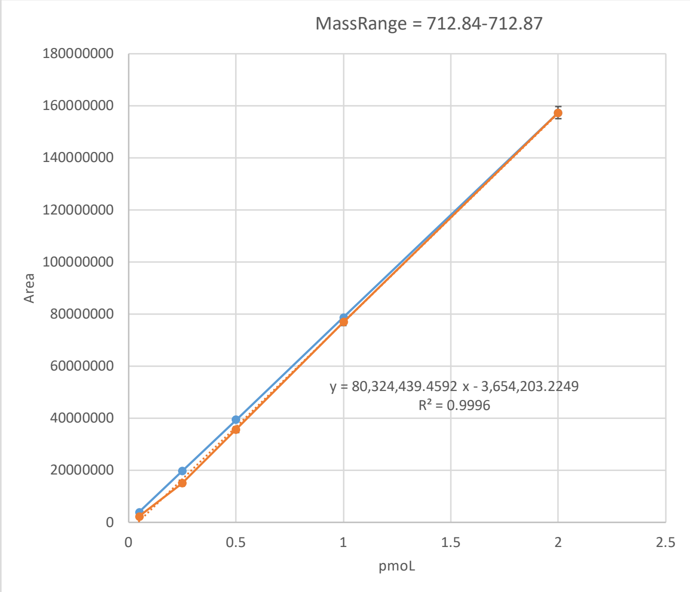

Supporting Information 7 (SI-7)

The raw data of the calibration curve of entry 4 NPY in LC-MS using YMC-Triart C8.

Sample solution: 50% acetonitrile/0.5% TFA, Matrix: 10 fmol/μL digested BSA

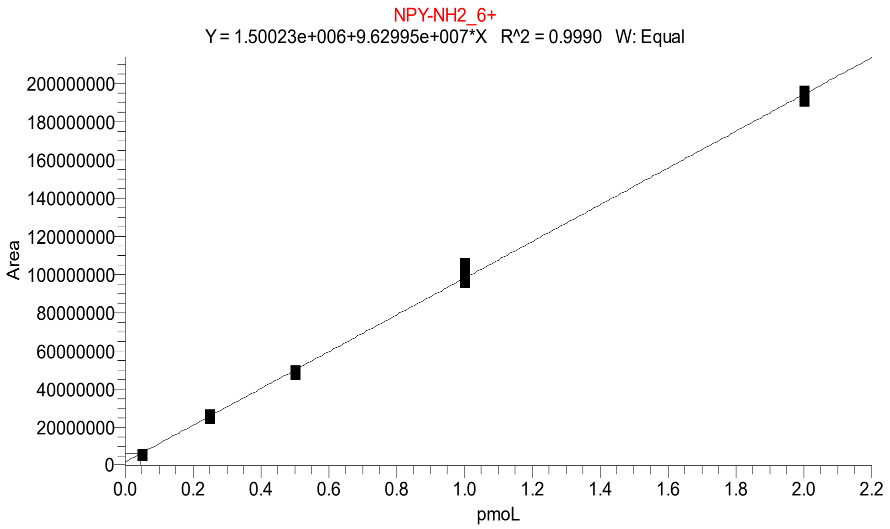

pmol	0.0500	0.2500	0.5000	1.0000	2.0000
run 1	5,958,059	26,290,241	49,976,892	106,203,465	191,836,528
run 2	5,646,309	25,771,693	47,908,604	98,950,655	191,066,184
run 3	5,757,907	26,579,344	49,424,093	97,028,111	196,022,792
run 4	5,729,540	25,133,530	49,593,576	100,864,203	193,003,463
run 5	5,229,032	24,545,452	48,239,358	96,182,477	194,254,474
Average	5664169.18	25664052.14	49028504.69	99845782.26	193236688.18
SD	240455.62	744872.77	806494.06	3567005.52	1762055.24
RSD(%)	4.25	2.90	1.64	3.57	0.91
SE	107535.02	333117.23	360675.11	1595213.36	788015.06
Ideal value	4830917	24154586	48309172	96618344	193236688
Bias(%)	17.25	6.25	1.49	3.34	0.00

Slope	96299482.14
Y-intercept	1500232.86
X-intercept	-0.0156

	Average	SD
R	0.999872	0.555323
R^2	0.999745	0.308383

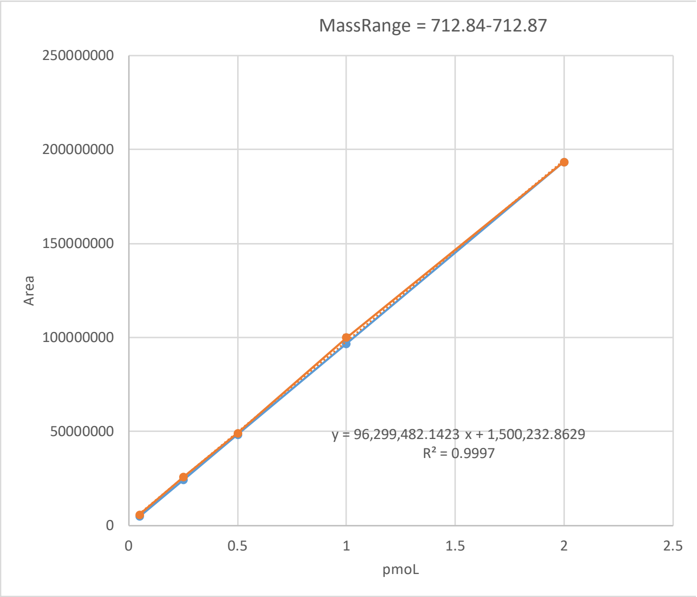

Supporting Information 8 (SI-8)

The raw data of the calibration curve of entry 5 NPY in LC-MS using YMC-Triart C8.

Sample solution: 50% acetonitrile/0.5%TFA, Matrix:10fmol/μL digested BSA+0.5pmol/μL orexin-B

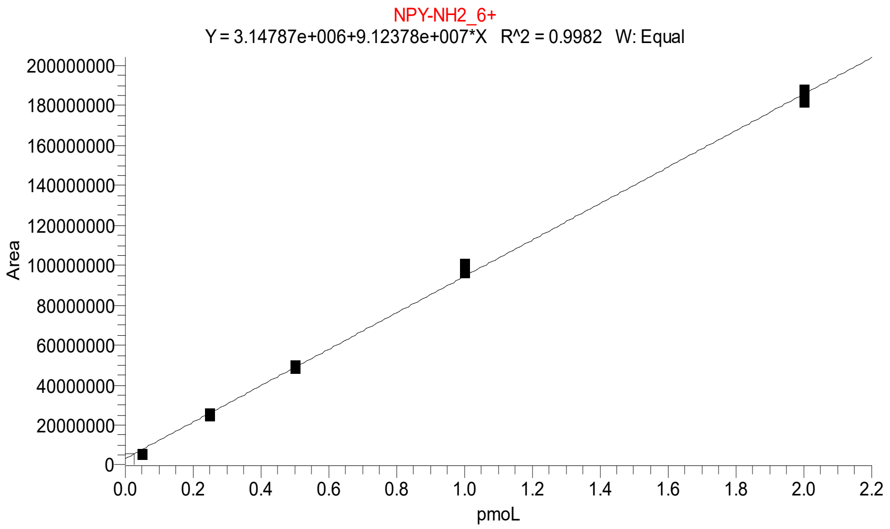

pmol	0.0500	0.2500	0.5000	1.0000	2.0000
run 1	5,627,869	25,172,972	49,120,680	96,396,203	182,219,944
run 2	5,746,266	25,756,127	50,189,079	98,538,027	182,803,507
run 3	5,556,831	25,774,547	49,067,571	98,474,773	182,251,890
run 4	5,577,659	24,620,331	49,188,634	100,848,603	181,806,813
run 5	5,695,441	25,371,713	48,848,730	99,381,324	188,180,298
Average	5640813.04	25339138.22	49282938.92	98727786.22	183452490.35
SD	71152.56	426337.27	467168.30	1446463.26	2385045.35
RSD(%)	1.26	1.68	0.95	1.47	1.30
SE	31820.39	190663.82	208924.02	646878.04	1066624.71
Ideal value	4586312	22931561	45863123	91726245	183452490
Bias(%)	22.99	10.50	7.46	7.63	0.00

Slope	91237847.08
Y-intercept	3147869.57
X-intercept	-0.0345

	Average	SD
R	0.999296	0.988281
R^2	0.998592	0.976698

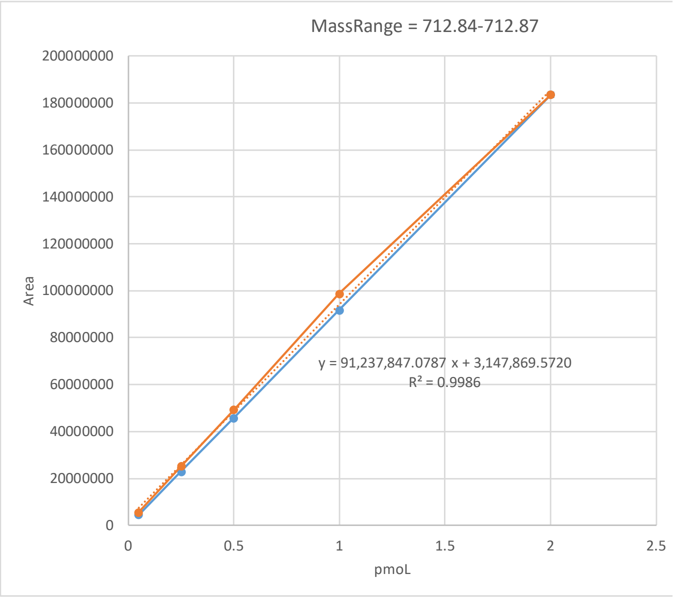

Supporting Information 9 (SI-9)

The raw data of the calibration curve of entry 6 NPY in LC-MS using YMC-Triart C8.

Sample solution: 35% acetonitrile/0.1%TFA, Matrix:10fmol/μL digested BSA+1.0pmol/μL orexin-B

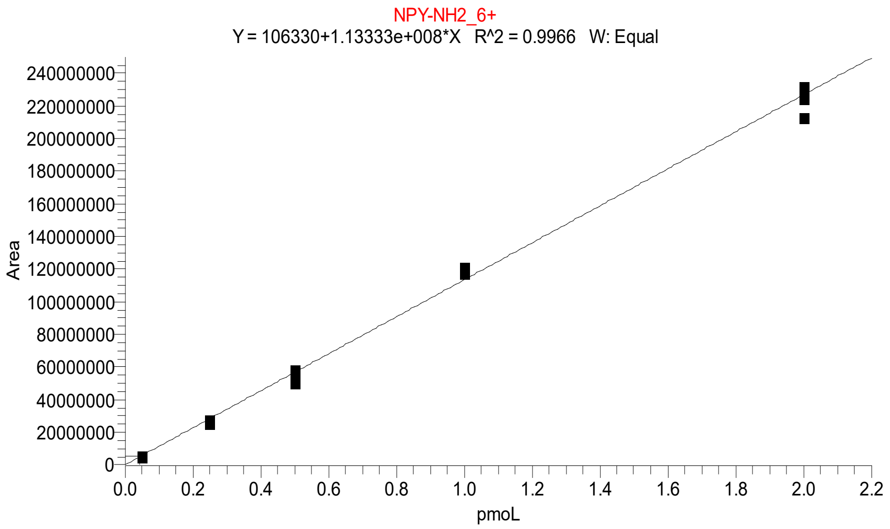

pmol	0.0500	0.2500	0.5000	1.0000	2.0000
run 1	5,214,614	25,982,564	49,746,601	117,089,893	231,291,575
run 2	5,262,943	27,281,869	56,776,140	119,960,502	226,973,569
run 3	5,254,777	25,441,940	56,719,448	120,110,382	225,438,163
run 4	5,333,810	27,002,400	58,465,962	121,154,139	224,376,830
run 5	4,919,327	25,064,403	58,076,328	120,610,016	212,438,091
Average	5197094.13	26154635.44	55956895.87	119784986.39	224103645.72
SD	144094.61	862033.77	3181440.38	1411061.21	6290365.95
RSD(%)	2.77	3.30	5.69	1.18	2.81
SE	64441.07	385513.22	1422783.39	631045.76	2813137.17
Ideal value	5602591	28012956	56025911	112051823	224103646
Bias(%)	−7.24	−6.63	−0.12	6.90	0.00

Slope	113333054.64
Y-intercept	106329.99
X-intercept	−0.0009

	Average	SD
R	0.999141	0.883769
R^2	0.998283	0.781048

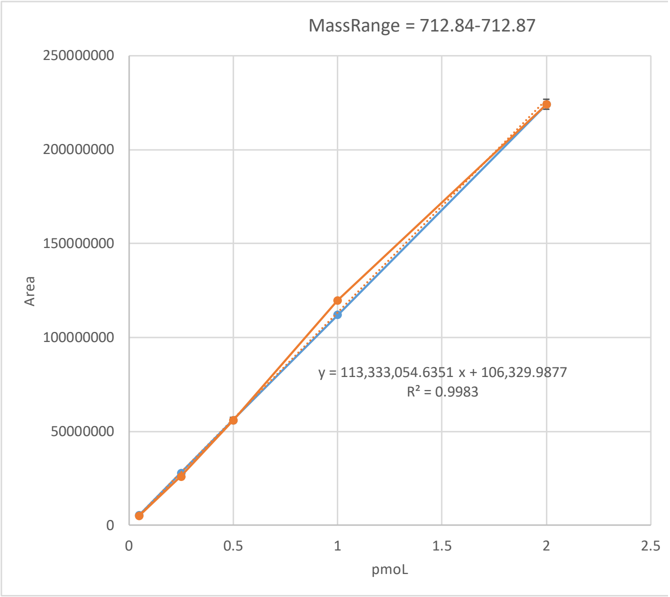

Supporting Information 10 (SI-10)

The raw data of the calibration curve of entry 7 NPY in LC-MS using YMC-Triart C8.
Sample solution: 35% acetonitrile/0.1%TFA, Matrix: 1.0 pmol/μL orexin-B only

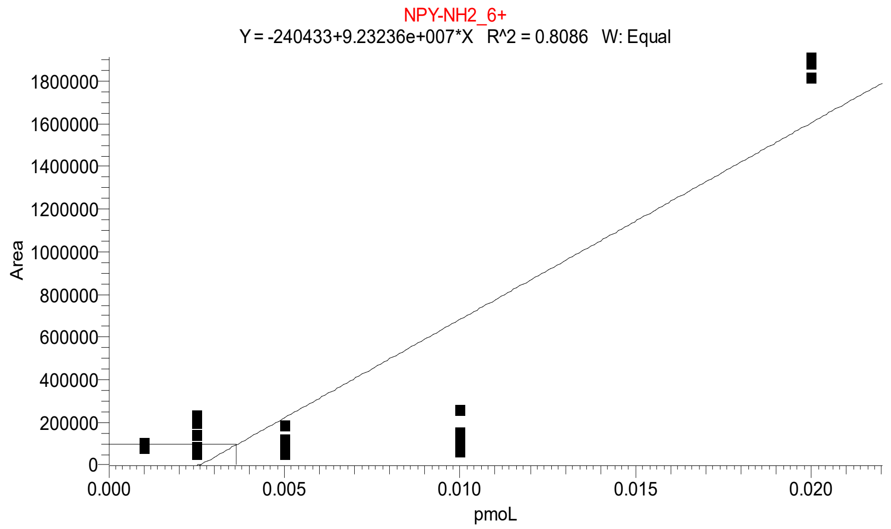

pmol	0.0010	0.0025	0.0050	0.0100	0.0200
run 1	95,571	231,604	185,846	258,394	1,909,577
run 2	82,921	200,302	122,742	154,581	1,818,852
run 3	84,698	140,734	89,226	115,194	1,883,228
run 4	101,866	86,822	56,819	106,471	1,885,598
run 5	98,675	51,056	50,942	64,152	1,885,594
Average	92746.34	142103.68	101115.14	139758.48	1876569.64
SD	7584.40	67446.30	49533.59	65911.90	30423.81
RSD(%)	8.18	47.46	48.99	47.16	1.62
SE	3391.85	30162.90	22152.09	29476.70	13605.94
Ideal value	93828	234571	469142	938285	1876570
Bias(%)	−1.15	−39.42	−78.45	−85.10	0.00

Slope	92323581.90
Y-intercept	−240432.92
X-intercept	0.0026

	Average
R	0.901439
R^2	0.812593

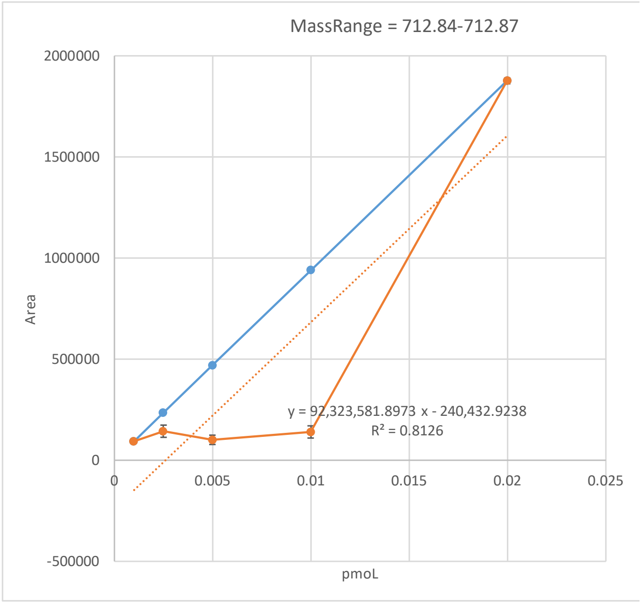

Supporting Information 11 (SI-11)

The raw data of the calibration curve of entry 8 NPY in LC-MS using YMC-Triart C8.

Sample solution: 35% acetonitrile/0.1%TFA, Matrix:10fmol/μL digested BSA 1.0pmol/μL orexin-B

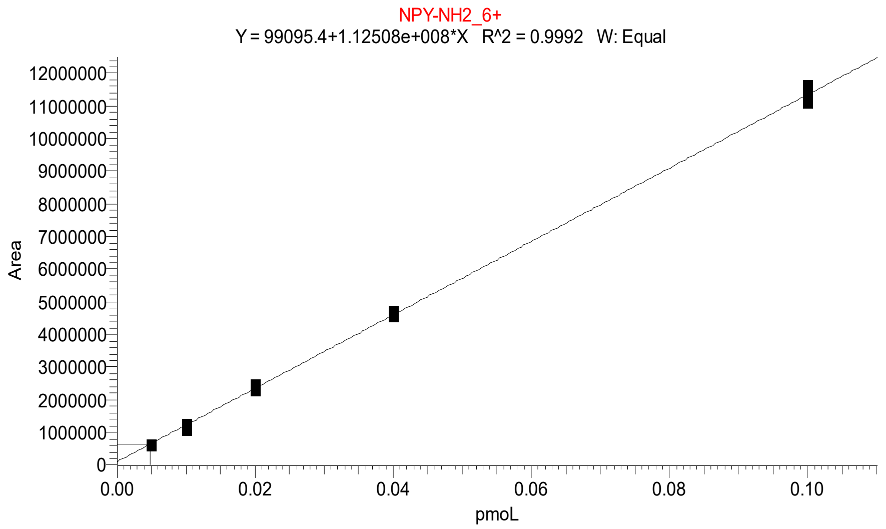

pmol	0.0050	0.0100	0.0200	0.0400	0.1000
run 1	640,962	1,278,583	2,452,413	4,686,883	11,236,089
run 2	606,295	1,267,468	2,307,427	4,555,776	11,493,135
run 3	653,525	1,252,076	2,279,434	4,663,105	11,653,077
run 4	639,365	1,228,029	2,344,665	4,594,333	11,114,902
run 5	614,441	1,092,739	2,363,082	4,741,919	11,161,726
Average	630917.71	1223778.99	2349404.15	4648403.35	11331785.61
SD	17669.94	67679.44	59123.97	66250.13	207064.43
RSD(%)	2.80	5.53	2.52	1.43	1.83
SE	7902.24	30267.17	26441.04	29627.96	92602.03
Ideal value	566589	1133179	2266357	4532714	11331786
Bias(%)	11.35	8.00	3.66	2.55	0.00

Slope	112507502.22
Y-intercept	99095.39
X-intercept	-0.0009

	Average
R	0.999976
R^2	0.999952

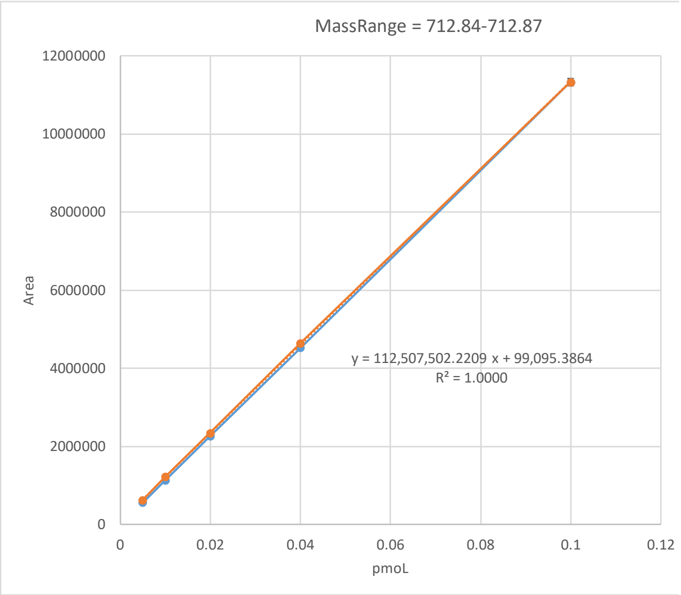

Supporting Information 12 (SI-12)

The raw data of the calibration curve of entry 9 NPY in LC-MS using YMC-Triart C8.

Sample solution: 35% acetonitrile/0.1%TFA, Matrix:10fmol/μL digested BSA 1.0pmol/μL orexin-B

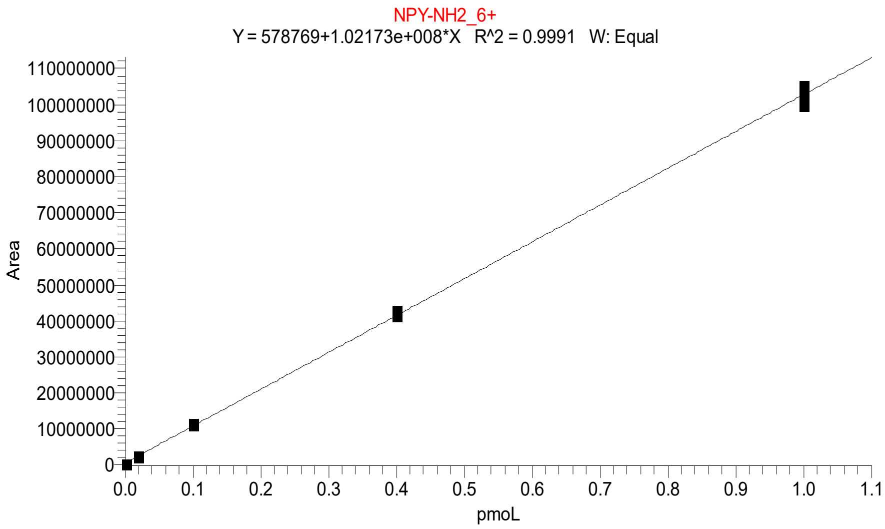

pmol	0.0020	0.0200	0.1000	0.4000	1.0000
run 1	224,190	2,313,740	11,005,083	42,834,674	100,512,263
run 2	243,205	2,293,097	11,417,490	41,745,782	103,662,214
run 3	240,682	2,326,356	11,531,564	43,055,131	99,468,071
run 4	245,265	2,231,280	11,552,833	41,244,863	103,443,176
run 5	233,513	2,218,718	11,260,255	41,541,238	105,163,855
Average	237370.98	2276638.14	11353444.95	42084337.57	102449915.95
SD	7693.12	43660.93	202792.33	723842.43	2119792.09
RSD(%)	3.24	1.92	1.79	1.72	2.07
SE	3440.47	19525.76	90691.49	323712.18	947999.84
Ideal value	204900	2048998	10244992	40979966	102449916
Bias(%)	15.85	11.11	10.82	2.69	0.00

Slope	102173366.62
Y-intercept	578768.72
X-intercept	-0.0057

	Average
R	0.999917
R^2	0.999834

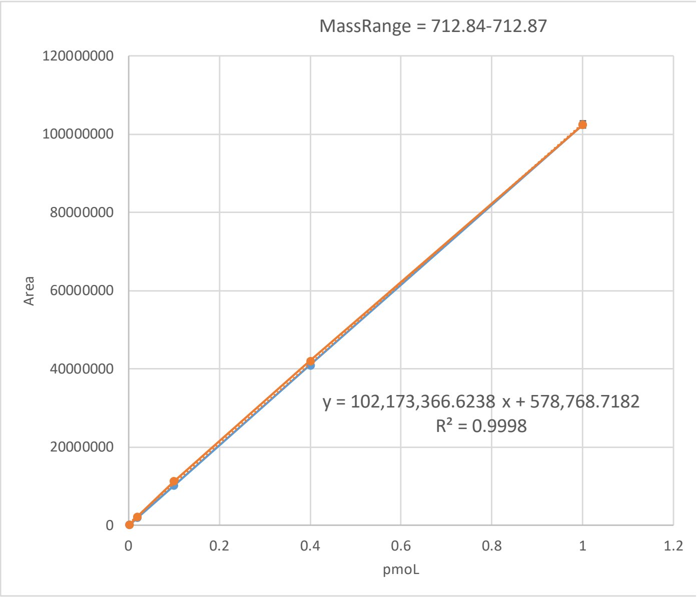

Supporting Information 13 (SI-13)

The raw data of the calibration curve of entry 10 NPY in bio-inert LC-MS using YMC-Triart C8. (35% acetonitrile/0.1%TFA, 10fmol/μL digested BSA 1.0pmol/μL orexin-B)

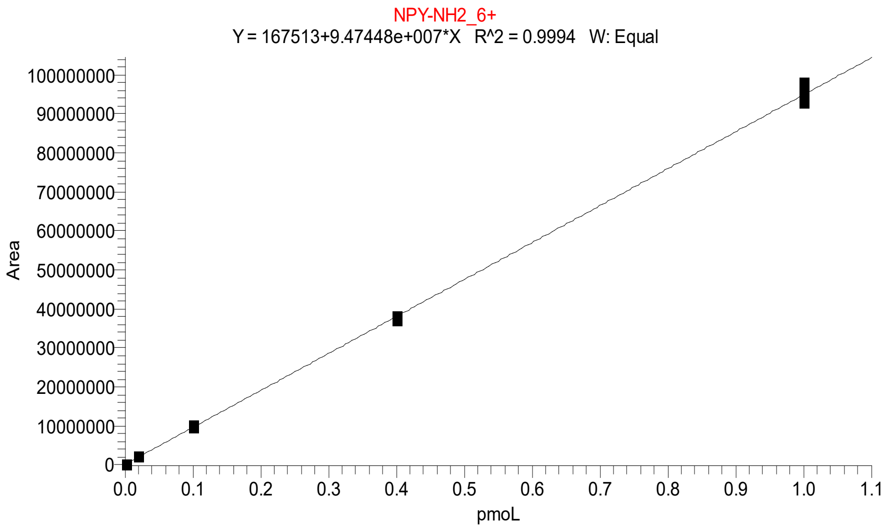

pmol	0.0020	0.0200	0.1000	0.4000	1.0000
run 1	229,756	2,200,235	9,553,737	37,606,960	94,083,560
run 2	222,288	2,144,496	9,963,242	37,088,628	94,832,567
run 3	215,171	2,210,460	9,892,971	38,172,354	92,767,894
run 4	225,616	2,211,961	10,099,760	37,345,850	95,660,998
run 5	236,202	2,263,369	10,198,871	37,783,122	97,985,859
Average	225806.77	2206104.24	9941716.24	37599382.61	95066175.77
SD	7059.80	37848.39	221121.57	370745.78	1742444.66
RSD(%)	3.13	1.72	2.22	0.99	1.83
SE	3157.24	16926.32	98888.57	165802.55	779244.94
Ideal value	190132	1901324	9506618	38026470	95066176
Bias(%)	18.76	16.03	4.58	−1.12	0.00

Slope	94744822.33
Y−intercept	167513.21
X−intercept	−0.0018

	Average
R	0.999971
R^2	0.999942

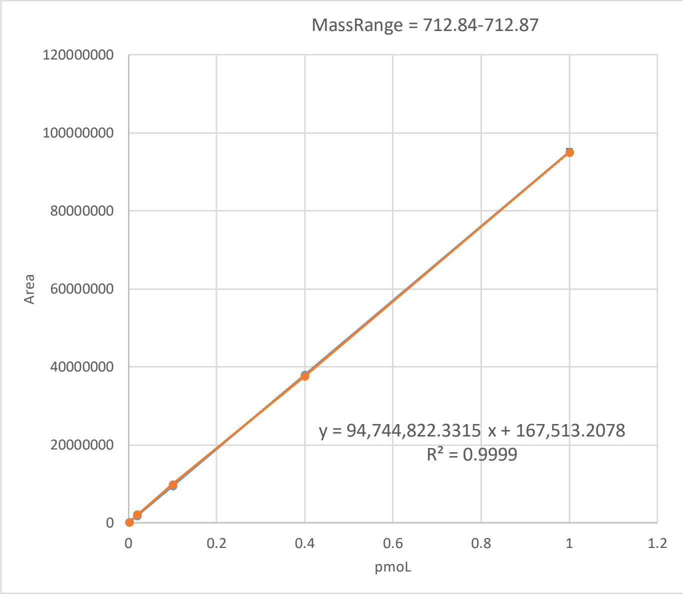

Supplement: Supplementary Data [file massspectrometry-9-1_A0087_s001.pdf]
